# An Intelligent Diagnosis Method of Brain MRI Tumor Segmentation Using Deep Convolutional Neural Network and SVM Algorithm

**DOI:** 10.1155/2020/6789306

**Published:** 2020-07-14

**Authors:** Wentao Wu, Daning Li, Jiaoyang Du, Xiangyu Gao, Wen Gu, Fanfan Zhao, Xiaojie Feng, Hong Yan

**Affiliations:** ^1^Department of Epidemiology and Health Statistics School of Public Health, Xi'an Jiaotong University, Xi'an, China; ^2^School of Public Health, Xi'an Jiaotong University, Xi'an, China; ^3^The First Affiliated Hospital, Xi'an Jiaotong University Health Science Center, Xi'an, Shaanxi 710061, China

## Abstract

Among the currently proposed brain segmentation methods, brain tumor segmentation methods based on traditional image processing and machine learning are not ideal enough. Therefore, deep learning-based brain segmentation methods are widely used. In the brain tumor segmentation method based on deep learning, the convolutional network model has a good brain segmentation effect. The deep convolutional network model has the problems of a large number of parameters and large loss of information in the encoding and decoding process. This paper proposes a deep convolutional neural network fusion support vector machine algorithm (DCNN-F-SVM). The proposed brain tumor segmentation model is mainly divided into three stages. In the first stage, a deep convolutional neural network is trained to learn the mapping from image space to tumor marker space. In the second stage, the predicted labels obtained from the deep convolutional neural network training are input into the integrated support vector machine classifier together with the test images. In the third stage, a deep convolutional neural network and an integrated support vector machine are connected in series to train a deep classifier. Run each model on the BraTS dataset and the self-made dataset to segment brain tumors. The segmentation results show that the performance of the proposed model is significantly better than the deep convolutional neural network and the integrated SVM classifier.

## 1. Introduction

The incidence of brain tumors increases with age [1]. This article focuses on gliomas in brain tumors. According to the location of the glioma, the cell type, and the severity of the tumor, the World Health Organization classifies the glioma into I~IV grades. Among them, Classes I and II are low-grade gliomas, and Classes III and IV are high-grade gliomas [2]. In order to facilitate doctors to accurately remove gliomas during surgery, Computed Tomography (CT), Magnetic Resonance Imaging (MRI), and Positron Emission Computed Tomography (PET) and other imaging techniques are commonly used in clinical treatment to brain image segmentation of the glioma area which helps the doctor to safely remove the tumor within the maximum range. At the same time, MRI has the characteristics of significant soft tissue contrast and can provide abundant physiological tissue information. In the clinical treatment of gliomas, MRI is usually used to diagnose gliomas preoperatively, intraoperatively, and postoperatively.

**Figure 1:**
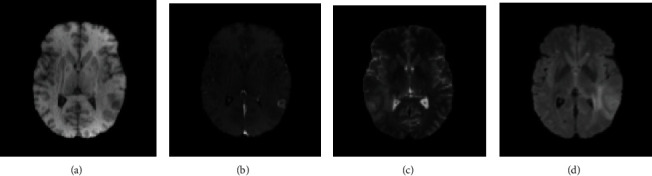
MRI of glioma: (a) T1-weighted, (b) postcontrast T1-weighted, (c) T2-weighted, and (d) FLAIR.

Glioma is a tumor composed of a necrotic core, a margin of tumor activity, and edema tissue. Multiple MRI sequences can be used to image different tumor tissues [3], as shown in Figure 1. At present, MRI imaging of gliomas generally has four modal sequences: T1-weighted, post-contrast T1-weighted, T2-weighted, and FLAIR. Different sequences reflect different glioma tissues [4]. The general FLAIR sequence is suitable for observing edema tissues, and the T1ce sequence is suitable for observing the active components of the tumor core.

MRI-based segmentation of gliomas and their surrounding abnormal tissues facilitates the doctor to observe the external morphology of each tumor tissue of the patient's glioma and also facilitates the doctor's imaging-based analysis and further treatment. Therefore, the segmentation of glioma is considered to be a first step in the MRI analysis of glioma patients. Because gliomas have different degrees of deterioration and contain multiple tumor tissue regions and brain MRI is a multimodal and many-layer three-dimensional scan image, manual segmentation of glioma regions requires a lot of time and manpower. In addition, manual segmentation is often based on the brightness of the image observed by the human eye for area segmentation, which is easily affected by the quality of the image generation and the personal factors of the tagger. It is prone to erroneous segmentation and segmentation of redundant areas. Therefore, in clinical practice, a fully automatic segmentation method with good segmentation accuracy for gliomas is needed. However, the problems in the study of automatic glioma segmentation methods are summarized as follows: (1) glioma is often distinguished in the image by the change in pixel intensity between the lesion area and surrounding normal tissues. Due to the presence of a gray-scale offset field, the intensity gradient between adjacent tumor tissues will be smoothed, resulting in blurred tumor tissue boundaries. (2) The structure of gliomas differs in size, shape, and position, making segmentation algorithms difficult to model. And because the growth position of glioma is not fixed, it is often accompanied by a tumor mass effect. This will cause the surrounding normal brain tissue to be compressed and change its shape, thereby generating irregular background information and increasing the difficulty of segmentation.

At present, computer-aided diagnosis technology based on machine learning has been widely used in medical image analysis in recent years [5–14]. Since the algorithm based on machine learning can train model parameters through various features of medical images and use the trained model to predict the extracted features, it can well solve the classification, regression, and aggregation in medical images. At the same time, the deep learning technology in machine learning can directly obtain high-dimensional features directly from the data and automatically adjust the model parameters through forward propagation and back-regulation algorithms, so that the performance of the model in related tasks can be optimized. Therefore, medical data processing of deep learning technology has developed into a research hotspot.

Brain tumor segmentation methods can be roughly divided into three categories: based on traditional image algorithms [15–20], based on machine learning [21–24], and based on deep learning [25–30]. In recent years, deep learning has become the method of choice for complex tasks due to its high accuracy. The convolutional neural network (CNN) proposed in [25] has made tremendous progress in the field of image processing. Therefore, the segmentation method based on the convolutional neural network is widely used in segmentation of lung nodules, retinal segmentation, liver cancer segmentation, and glioma segmentation [26]. Many scholars have begun to apply CNN in deep learning to segmentation of gliomas. Reference [31] proposes a brain cancer segmentation method based on dual-path CNN. Reference [32] trained two CNNs to segment high-grade gliomas and low-grade gliomas. Reference [33] proposed a two-channel three-dimensional CNN for glioma segmentation.

This paper mainly studies the segmentation method of glioma based on the deep learning method, aiming at automatically and accurately segmenting the glioma region from the brain MRI through the deep learning algorithm. For the task of glioma segmentation, this paper proposes a DCNN-F-SVM deep classifier. The main research contents of this article are as follows:

1. A new depth classifier is proposed. The classifier is composed of a deep convolutional neural network and an integrated SVM algorithm. First, CNN was trained to learn the mapping from image space to tumor label space. The predicted labels in CNN together with the test images were input into an integrated SVM classifier. In order to make the results more accurate, we deepened the classification process and iterated these two steps again to form the framework of the next CNN-SVM in series
2. The traditional segmentation method is to use the training set to train a suitable classifier, and then test the set for verification. The method proposed in this study is completely different from the traditional method. The proposed model mainly includes three stages: one is preprocessing, feature extraction, and training CNN and SVM. The second is to test and generate the final segmentation results. The third is to deepen the order of our CNN-SVM cascade classifier through an iterative step
3. Apply the proposed model to public datasets and self-made datasets for evaluation. Compared with the segmentation performance of CNN and SVM alone, the superiority of the proposed model can be reflected in various evaluation indexes

## 2. Related Works

### 2.1. Process of Brain Tumor Segmentation Algorithm Based on Deep Learning

In the currently proposed glioma segmentation method, the segmentation results of traditional image processing algorithms rely heavily on manual intervention, and a priori constraints are required to ensure the segmentation effect, resulting in poor robustness and low efficiency of the method. The glioma segmentation method based on machine learning needs to manually select the features of the image, so that the segmentation effect of this type of method depends on the artificial features, and the generalization ability of the segmentation algorithm itself is weak.

**Figure 2:**
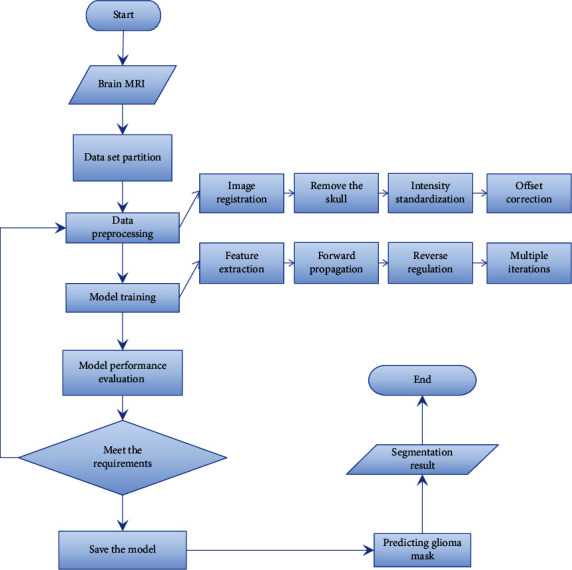
Flow chart of glioma segmentation algorithm based on deep learning.

The glioma segmentation method based on deep learning can automatically extract image features through the neural network model and segment the glioma region. Therefore, the shortcomings of strong prior constraints and manual intervention in the above method are overcame. The automation and robustness of the segmentation algorithm are improved, and good segmentation results can be achieved in large-scale complex glioma segmentation scenarios.  Figure 2 is the flow of glioma segmentation algorithm based on deep learning. The process can be described as follows: first, obtain the MRI of the patient's brain and use it as the input data of the algorithm; then, divide the input data into the training set, verify the set, and test the set. At the same time, due to factors such as noise and uneven intensity in the original brain MRI, the divided data needs to be preprocessed. Commonly used glioma image preprocessing methods include image registration, skull removal, intensity standardization, and offset correction. Next, use the preprocessed input data to train the deep learning model. During the training process, the deep model will automatically perform feature extraction, and add the extracted features to the designed model structure for forward propagation. At the same time, the multiregion mask of glioma is used as a label to calculate the loss value, so that the model parameters are reversely adjusted in multiple iterations to achieve the purpose of optimal model performance. Then, at the end of each iteration, different evaluation indicators are used to evaluate the performance of the model, and the models that meet the conditions of the indicators are saved. Finally, the highly evaluated model is used to segment the test set data to obtain the final glioma segmentation results.

### 2.2. A Deep Brain Tumor Feature Generation Method

CNNs are well-known practical models in the field of deep learning, and their innovative ideas stem from the processing of human brain nerves. The perceptron model proposed in 1980 is considered to be the original model of convolutional neural networks. The perceptron model is a classic model in the field of machine learning, but this model also has great shortcomings and cannot solve XOR problems well. On this basis, reference [34] proposed the LeNet model, which has multiple convolutional layers, and each layer is a fully connected model trained using the back propagation algorithm [35]. Reference [36] proposed an artificial neural network called displacement invariance and studied the parallel structure of the convolutional neural network. However, these models are limited by experimental data and hardware conditions. Therefore, it is not suitable for complex tasks such as object detection and scene classification. In order to solve some problems in the training process of convolutional neural networks, Krizhevsky et al. proposed the AlexNet model [37]. In order to solve the overfitting problem of convolutional neural networks, the model proposes local convolution and Relu technologies, and the overfitting problem is well solved.

**Figure 3:**
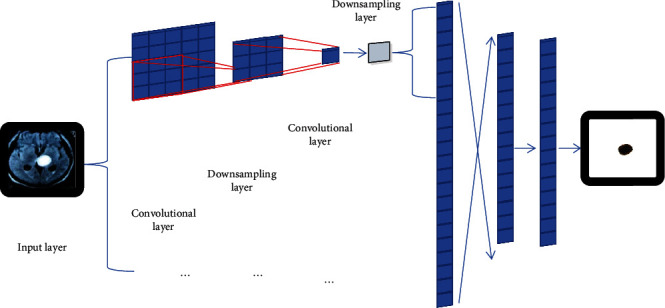
LeNet convolutional neural network structure.

CNN is essentially a multilayer perceptron and a multilayer neural network, and there is an obvious sequence between these layers, which is composed of an input layer, a hidden layer, and an output layer. There can be multiple hidden layers, and each layer is composed of multiple two-dimensional planes. Each plane contains multiple neurons, and the hidden layer consists of a convolution layer, a downsampling layer, and a fully connected layer. The convolution layer and the downsampling layer appear alternately and can have multiple layers, and the fully connected layer can also have multiple layers. The network structure of the traditional convolutional neural network LeNet is shown in Figure 3.

In the convolution layer, the feature maps output by the previous layer are convolved by the learned convolution kernel, and the corresponding partial derivatives are input into the activation function together to form an output feature maps. The downsampling layer is used for feature selection to select representative features. The fully connected layer is a neural network layer whose role is to map two-dimensional distributed features into feature vectors for better classification. The output layer is a simple classification layer, usually using logistic regression for classification. Here, we use the Softmax classifier for classification.

The activation function usually selects a nonlinear function to better fit the nonlinear model. Selecting the activation function needs to consider its monotonicity and derivability. Common activation functions are shown as follows:

1. Relu function: *f*(*x*) = max(0, *x*)
2. Softplus function: *f*(*x*) = log(1 + *e*^*x*^)

**Figure 4:**
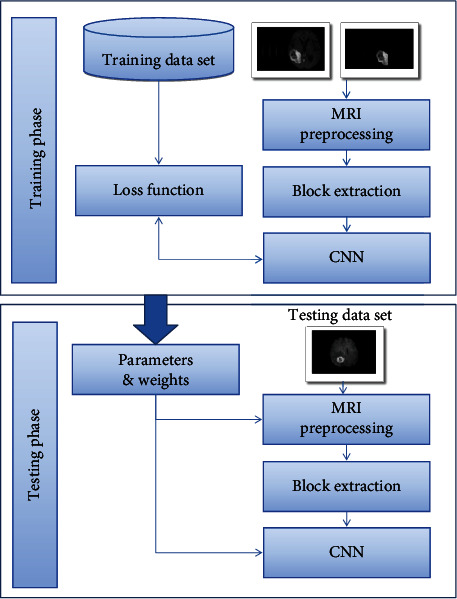
CNN flow chart.

The CNN model structure is simpler and easier to expand than the neurocognitive machine. In the neurocognitive machine, the downsampling layer and the convolutional layer alternate to form the function of feature extraction and abstraction, while in the convolutional neural network, the convolutional layer and the downsampling layer alternate, and their functions are similar. The convolution operation simplifies feature extraction, the excitation function replaces multiple nonlinear functions of the neurocognitive machine, and the pooling operation is also simpler. The CNN algorithm flow is shown in Figure 4.

### 2.3. Introduction of Brain Tumor Dataset

The BraTS Challenge held in 2012 provided a brain MRI dataset with both low-grade gliomas and high-grade gliomas. The dataset provides MRI of multiple patients and provides a multiregion glioma segmentation ground truth for each patient. Among them, ground truth is the result of fusion of 20 segmentation algorithms and then manually labeled by multiple human experts. Every BraTS competition will provide a public dataset of gliomas. However, the glioma dataset provided since BraTS17 has been significantly different from the dataset provided before 2016. The dataset used between BraTS14 and BraTS16 contains images of gliomas before and after surgery, which leads to confusing glioma segmentation criteria in the dataset and does not have the conditions to be true segmentation criteria. Therefore, the datasets between BraTS14 and BraTS16 are no longer used in the games after BraTS17. The BraTS18 dataset is based on the BraTS17 dataset with the addition of the TCIA glioma dataset. The TCIA glioma dataset includes 262 high-grade glioma patient images and 199 low-grade glioma patient images. This dataset contains the MRI and ground truth of 543 glioma patients and is currently the most standard glioma segmentation dataset. The details of the datasets in the BraTS competition datasets over the years are shown in Table 1.

**Figure 5:**
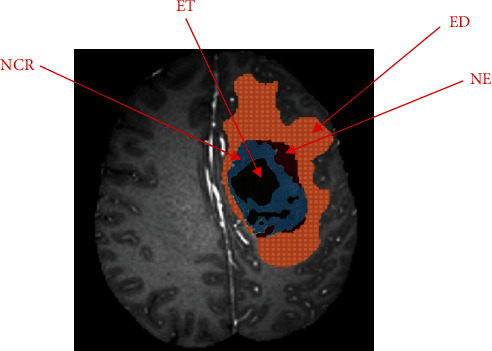
Tumor area division of glioma.

As shown in Figure 5, gliomas are generally divided into four tumor regions, namely, edema around the tumor (ED), nonenhanced tumor core (NET), enhanced tumor core (ET), and necrotic core (NCR). Among them, ED, NET, and NCR are real glioma tumor tissues. The enhancement of the tumor core is to facilitate the observation of the tumor core.

### 2.4. Evaluation Method of Segmentation Result

The common evaluation methods for evaluating the performance of each model in the field of image segmentation are shown in Table 2.

In addition to the above evaluation indicators, there are indicators such as Hausdorff Li and positive predictive value. The most commonly used are DSC and sensitivity.

## 3. Introduction of DCNN-F-SVM Model

**Figure 6:**
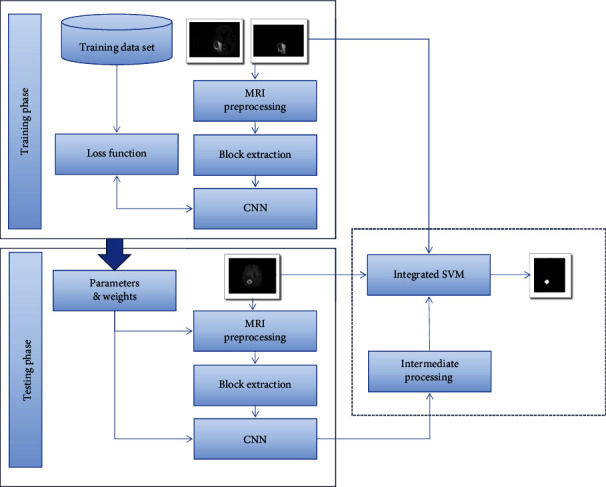
The proposed model flow chart.

This study proposes a brain tumor segmentation model based on convolutional neural network fusion SVM.  Figure 6 is the model flow chart.

**Figure 7:**
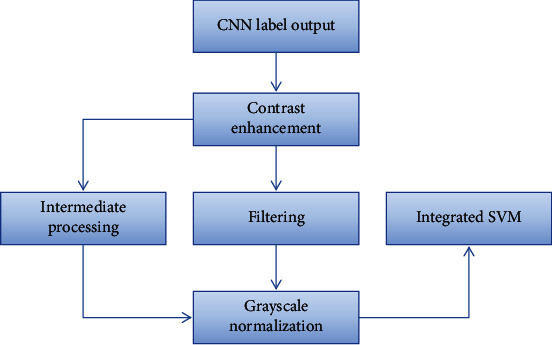
Schematic diagram of intermediate processing.

The proposed model segmentation of brain tumor images can be divided into two parts: one is preprocessing, feature extraction, and training CNN and SVM; the other is testing and generating the final segmentation results. It can be divided into 3 stages. In the first stage, CNN and integrated SVM are trained to obtain the mapping from the gray image domain to the tumor label domain. In the second stage, the labeled output of CNN and the test image are input into the integrated SVM classifier. In the third stage, an iterative step is used to connect the CNN and the integrated SVM classifier, which increases the number of layers. In order to select the optimal feature, an intermediate processing step is added to the model, as shown in Figure 7.

Grayscale, mean, and median are used to represent each pixel. These features are used to train CNN to obtain a nonlinear mapping between input features and labels. In the testing stage, an aggregated SVM classifier is independently trained using the aggregated CNN label map and the same features as before.

An iterative classification process is applied to the preprocessed input image. First, CNN classifies the pixels in the key area, thus generating a kind of presegmentation, which will be sent to the integrated SVM classifier. Then, a Region Of Interest (ROI) on presegmentation will be generated. In addition to presegmentation, classification based on integrated SVM will be performed on this ROI. After that, the integrated SVM explores the neighborhood of the CNN output. Use CNN to classify the marked ROI again. Repeat the above steps to further refine the segmentation results.

## 4. Simulation Experiment

### 4.1. Experiment-Related Instructions

The experimental dataset used in this study includes the public dataset and the self-made dataset. The comparison models are SVM, CNN, and DCNN-F-SVM. In the setting of experimental parameters, set the window size to 5, *σ*=0.1, and *C* = 1000. The public dataset used is the BraTS18 dataset. The self-made dataset is the clinical MRI images of 26 patients. The evaluation index used in the experiment is DSC, sensitivity, and specificity. The description of the experimental software and hardware environment is shown in Table 3.

### 4.2. Public Dataset Experiment

After the model training is completed, the test set can be predicted by the model to obtain the glioma segmentation result obtained by the model segmentation. In the test set divided by three-fold cross-validation, the evaluation index pair of each model on the BraTS18 dataset is shown in Table 4. The data in the table fully shows that the proposed model has better tumor segmentation performance than SVM and CNN. Compared with SVM, the proposed algorithm has improved by 8.3%, 9.7%, and 1.4% on the three indicators: DSC, sensitivity, and specificity; compared with CNN, the proposed algorithm has three indicators: DSC, sensitivity, and specificity, increased by 4.7%, 2.6% and 0.2%, respectively.

### 4.3. Self-Made Data Experiment

In this section, clinical MRI images of 26 patients were collected, and brain tumors were trained and segmented using three models, and the experimental results were given. Tables 5 and 6 show the segmentation results of CNN and DCNN-F-SVM for 26 patients, respectively.

Among the index values shown in Table 5, the DSC values are generally distributed around 0.86 and have an up and down floating error of about 0.18. The sensitivity values are generally distributed around 0.89 and have a floating error of about 0.14. The specificity values are generally distributed around 0.95 and have an up and down floating error of about 0.11.

Among the index values shown in Table 6, the DSC value is generally distributed around 0.89, and there is an upward and downward floating error of about 0.15. The sensitivity values are generally distributed around 0.91, and there is about 0.12 up and down floating error. The specificity value is generally distributed around 0.96, and there is about 0.09 up and down floating error.

Table 7 shows the DSC, specificity, and sensitivity values of the three methods. The proposed DCNN-F-SVM has increased in comparison with CNN and SVM used independently, in which the three indicators in the table (DSC, sensitivity, and specificity) are 3.5%, 2.6%, and 3.2% higher compared to those of SVM and 1.6%, 0.9%, and 2.4% higher compared to those of CNN. The proposed model can indeed improve the segmentation performance.

## 5. Conclusion

The diagnosis of brain diseases requires accurate diagnosis without deviation. Any misdiagnosis will cause irreparable losses. The incidence of brain tumors in brain diseases has been high, and the number of patients has increased year by year. This has also increased the workload of medical personnel in this field to a certain extent. An accurate and efficient method of brain tumor image segmentation needs to be urgently proposed, which has solved the increasing demand. Based on this background, this paper proposes a depth classifier to improve the segmentation accuracy and achieve automatic segmentation without manual intervention. The classifier is mainly composed of DCNN and integrated SVM connected in series. The implementation of the model is divided into three stages. In the first stage, a deep convolutional neural network is trained to learn the mapping from the image space to the tumor marker space. In the second stage, the predicted labels obtained from the deep convolutional neural network training are input into the integrated support vector machine classifier together with the test images. In the third stage, a deep convolutional neural network and an integrated support vector machine are connected in series to train a deep classifier. The simulation implementation verified the superiority and effectiveness of the proposed model. However, the proposed model still has shortcomings such as long calculation time. How to optimize the algorithm and shorten the running time will be the next research content.

## Figures and Tables

**Table 1:** Introduction of BraTS dataset over the years.

Dataset	Date	Total number of samples			
		Training set	Validation set	Test set	Total
BraTS12	2012	30	10	25	65
BraTS13	2013	30	10	25	65
BraTS14	2014	40	10	25	65
BraTS15	2015	274	—	110	384
BraTS16	2016	274	—	191	465
BraTS17	2017	210	46	146	412
BraTS18	2018	285	67	191	543

**Table 2:** The description of the adopted indices.

Index	Expression/description
True Positive (TP)	TP indicates that the model predicts a glioma region, and the doctor marks pixels that are also glioma regions
False Positive (FP)	FP means pixels predicted by the model as the glioma area are actually the background area
True Negative (TN)	TN indicates that the model predicted as the background area is actually the pixel of the background area
True Negative (TN)	FN means pixels predicted by the model as the background area are actually as the tumor area
Dice Similarity Coefficient (DSC)	DSC = 2TP/FP + 2TP + FN
Sensitivity	Sens = TP/TP + FN
Specificity	Spec = TN/TN + FP

**Table 3:** Experimental environment description.

Hardware configuration		Software configuration	
Configuration item	Configuration parameter	Configuration item	Configuration parameter
Operating system	Ubuntu 14.04	Development environment	PyCharm
CPU	AMD A8-5600K	Programming language	Python
RAM	16.0GB	Image algorithm library	OpenCV
Video memory	479 MB	Deep learning algorithm library	TensorFlow

**Table 4:** Evaluation index of each model.

Model	DSC	Sensitivity	Specificity
SVM	0.8268	0.8306	0.9845
CNN	0.8556	0.8876	0.9962
DCNN-F-SVM	0.8958	0.9110	0.9982

**Table 5:** Evaluation data of 26 patients with brain tumor segmentation using the SVM model.

Number	DSC	Sensitivity	Specificity	Number	DSC	Sensitivity	Specificity
1	0.8801	0.9020	0.9563	14	0.8695	0.8896	0.9411
2	0.8768	0.8963	0.9368	15	0.8753	0.8976	0.9520
3	0.8893	0.9158	0.9605	16	0.8536	0.8729	0.9264
4	0.8682	0.8910	0.9482	17	0.8463	0.8667	0.9118
5	0.8926	0.9089	0.9795	18	0.8831	0.9053	0.9786
6	0.8796	0.8998	0.9385	19	0.8920	9107	0.9632
7	0.8859	0.9096	0.9543	20	0.8697	0.8896	0.9408
8	0.8633	0.8859	0.9386	21	0.8787	0.9006	0.9602
9	0.8828	0.9010	0.9715	22	0.8811	0.9120	0.9632
10	0.8989	0.9157	0.9634	23	0.8980	0.9234	0.9728
11	0.9003	0.9236	0.9726	24	0.8479	0.8752	0.9388
12	0.8429	0.8695	0.9367	25	0.8256	0.8610	0.9286
13	0.8396	0.8600	0.9302	26	0.8694	0.8887	0.9385

**Table 6:** Evaluation data of 26 patients with brain tumor segmentation using the DCNN-F-SVM model.

Number	DSC	Sensitivity	Specificity	Number	DSC	Sensitivity	Specificity
1	0.8923	0.9220	0.9663	14	0.8956	0.9222	0.9785
2	0.8867	0.9063	0.9368	15	0.8896	0.9185	0.9669
3	0.9091	0.9193	0.9702	16	0.8876	0.9104	0.9678
4	0.8782	0.9014	0.9588	17	0.8782	0.9086	0.9585
5	0.9026	0.9289	0.9795	18	0.9020	0.9103	0.9786
6	0.8998	0.9098	0.9405	19	0.9023	0.9123	0.9752
7	0.9056	0.9196	0.9743	20	0.8885	0.9116	0.9600
8	0.9030	0.9229	0.9696	21	0.8963	0.9205	0.9696
9	0.8927	0.9110	0.9711	22	0.9004	0.9287	0.9745
10	0.9126	0.9289	0.9806	23	0.9102	0.9258	0.9798
11	0.9185	0.9298	0.9885	24	0.8763	0.9115	0.9598
12	0.8789	0.9110	0.9605	25	0.8689	0.9088	0.9469
13	0.8825	0.9168	0.9693	26	0.8996	0.9305	0.9797

**Table 7:** Evaluation indexes of the segmentation results of the three models.

Method	DSC	Sensitivity	Specificity
SVM	0.8705	0.9001	0.9586
CNN	0.8869	0.9152	0.9657
DCNN-F-SVM	0.9010	0.9236	0.9889

## Data Availability

The labeled dataset used to support the findings of this study are available from the corresponding author upon request.
